# Account of Diseases in an Eastern District of London, from January 20, to February 20, 1803

**Published:** 1803-03-01

**Authors:** 


### *Account of Diseases in an Eastern District of London, from January* 20, *to February* 20, 1803.

| | |
|---|---|
| ACUTE DISEASES. | |
| Pneumonia | 12 |
| Typhus | 4 |
| Rheumatismus Acutus | 7 |
| Dysenteria | 3 |
| CHRONIC DISEASES. | |
| Dyspnœa | 17 |
| Tussis cum Dyspnœa | 24 |
| Catarrhus | 8 |
| Raucedo | 5 |
| Hæmoptoe | 3 |
| Phthisis Pulmonalis | 2 |
| Ascites | 4 |
| Hydrops Pectoris | 3 |
| Epistaxis | 2 |
| Hæmorrhagia Intestinalis | 2 |
| Enterodynia | 5 |
| Menorrhagia Difficilis | 2 |
| Amenorrhœa | 5 |
| Vertigo | 4 |
| Paralysis | 1 |
| Hypochondriasis | 2 |
| Rheumatismus Chronicus | 20 |
| PUERPERAL DISEASES. | |
| Peritonitis | 3 |
| Menorrhagia Lochialis | 3 |
| Abscessus Mammæ | 2 |
| Ephemera | 4 |
| INFANTILE DISEASES. | |
| Ophthalmia | 4 |
| Ophthalmia Purulenta | 3 |
| Febris Mesenterica | 2 |
| Aphthæ | 3 |

The List of Diseases, occurring in the present month, bears a considerable resemblance to that of the last. Complaints of the chest, as is usual at this season of the year, form a considerable part of it. Peripneumonies and catarrhal affections have been very general; in most instances they have been tedious, and in some fatal. In cases of the more acute kind, venæsection was highly necessary, and in some instances a repetition of it was attended with advantage. This was particularly indicated by the patients being troubled with a constant cough, a considerable degree of pain in the chest, and a deficiency of expectoration. A diminution of the pain and difficulty of of breathing, with an abatement of the cough, and an alleviation of all the febrile symptoms, in general succeeded the use of the lancet. Expectoration also became more free, and the discharge of a quantity of viscid mucus was accompanied by a more easy respiration. In those instances, where the perpetual irritation of coughing prevented the patient's sleeping, if the expectoration were tolerably free, and if venæsection and blistering had been premised, an anodyne was given at night with apparent advantage.

The opium, combined with antimony, or given in the form of pulv. ipec. comp. generally succeeded best; or, in the latter stage of the disease, the addition of a few drops of tinct. scill seemed to prevent that check of expectoration which is sometimes feared as a consequence of the unguarded use of opium.

